# The “oral-gut axis” transmission of microorganisms in colorectal cancer: Insights from *Peptostreptococcus’* perspective

**DOI:** 10.1080/21505594.2026.2673632

**Published:** 2026-05-26

**Authors:** Yuhong Zhu, Simin Luo, Yuanke Luo, Zhanli Guo, Yifang Jiang, Qiong Ma, Xi Fu, Chuan Zheng, Fengming You, Qixuan Kuang, Xueke Li

**Affiliations:** a TCM Regulating Metabolic Diseases Key Laboratory of Sichuan Province, Hospital of Chengdu University of Traditional Chinese Medicine, Chengdu, Sichuan, China; b Institute of Oncology, Chengdu University of Traditional Chinese Medicine, Chengdu, Sichuan, China; c Oncology Teaching and Research Department, Chengdu University of Traditional Chinese Medicine, Chengdu, Sichuan, China

**Keywords:** Oral-gut axis, colorectal cancer, *Peptostreptococcus*, underlying mechanism, biomarker

## Abstract

Colorectal cancer (CRC) is linked to gut microecological imbalances, and increasing evidence suggests that oral-gut microecosystem aberrant interactions also significantly contribute to CRC pathogenesis. The emerging “oral-gut axis” offers novel perspectives into the cross-organ microbial regulation. Notably, the oral bacterium *Peptostreptococcus* exhibits spatiotemporal specificity during CRC progression and may modulate gut microecology and CRC via this axis. This review examines the oral-gut microecological interplay and teases apart the multidimensional associations between *Peptostreptococcus* (e.g. *Peptostreptococcus stomatis*, *Peptostreptococcus anaerobius*) and CRC, including their heterogeneity across CRC patient groups and their dynamic evolution and spatial distribution during the “adenoma-carcinoma” sequence. It summarizes mechanisms whereby *Peptostreptococcus* influences CRC by promoting tumor cell proliferation, inducing epithelial–mesenchymal transition, and reshaping the tumor microenvironment. It also categorizes the key technologies in this field. Furthermore, this review highlights *Peptostreptococcus’s* potential as a CRC biomarker and therapeutic target, proposing future intervention strategies targeting oral-derived microbes to stimulate further research.

## GRAPHICAL ABSTRACT

**Figure:**
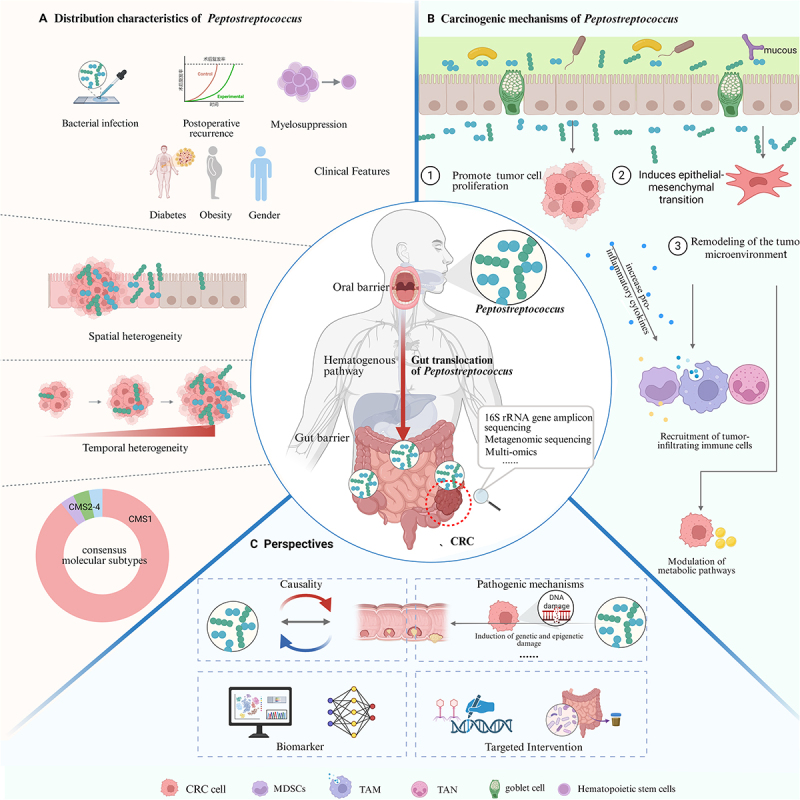

## Introduction

The “oral-gut axis” is an emerging critical dimension of human health. With the advent of next-generation sequencing (NGS) technology in the twenty-first century, researchers have transcended the constraints of traditional static single-site microbial studies and started to systematically explore cross-organ microbial associations. Within this framework, the theoretical system of the “oral-gut axis” has progressively formed: in 2017, Acharya et al. [1] initially explored the “oral-gut axis” based on the hypothesis that oral microorganisms, their metabolites, and inflammatory factors might traverse a compromised intestinal barrier. Subsequently, in 2019, Du Teil Espina et al. [2] formally established the concept of the oral-gut microbiome axis. In 2021, Park et al. [3] elucidated for the first time the bidirectional regulatory mechanism of the oral-gut microbiome axis in gastrointestinal diseases and cancers, suggesting that oral pathogens may promote colorectal cancer (CRC) by disrupting the intestinal barrier and altering the tumor microenvironment (TME).

CRC has become the third most prevalent malignant digestive tumor with the second-highest fatality rate worldwide [4,5]. Its development is influenced by a complex interplay of genetic and environmental factors, with microbes playing a crucial role. Common oral microorganisms, such as *Peptostreptococcus*, *Streptococcus*, and *Actinomyces*, exhibit pervasive translocation along the gastrointestinal tract in both healthy and diseased populations [6]. In contrast, *Fusobacterium nucleatum* (*F. nucleatum*) has been extensively documented as an occasional transmitter implicated in CRC progression through mechanisms such as promoting tumor immune evasion and chemoresistance [7,8]. Recently, *Peptostreptococcus*, another opportunistic pathogen, has also been selectively enriched in the fecal and mucosal microbiota of CRC patients [9]. This Gram-positive anaerobe often co-aggregates with *Fusobacterium* and *Porphyromonas* in the oral cavity [10] and is also present in the upper respiratory, intestinal, and female reproductive tracts. Concurrently, our research indicates that *Peptostreptococcus* may significantly influence CRC progression through the “oral-gut axis”. Compared to the well-systematized elucidation of key oral microorganisms like *F. nucleatum* in CRC, although *Peptostreptococcus* exhibits notable clinical associations with CRC, its interactions with the gut microbiota and the mechanisms by which it contributes to tumor progression have not been comprehensively integrated. Therefore, this review will take *Peptostreptococcus* as an entry point, considering both its commonalities and distinctions with other pivotal oral microorganisms. Within the core framework of the “oral-gut axis”, we will analyze the relationship between microbial translocation along this axis and CRC, summarize the latest research on *Peptostreptococcus* and CRC, and discuss its potential applications and challenges in the clinical diagnosis and treatment of CRC. We aim to enhance the understanding of the “oral-gut axis” and provide a new perspective for microbiome-based interventions in digestive diseases.

## Microecological interaction between the oral cavity and the intestine

The oral cavity and the intestinal tract, as two ends of the digestive tract, are anatomically and neurologically related, forming part of the same mucosal immune system [11]. From a microbiological perspective, they are also the body’s largest microbial habitats, collaborating in the “wetland system” (i.e. the microecology of the digestive tract) to maintain ecological balance and support host physiology [3,12]. Advances in multi-omics have expanded our understanding of the microbial communities, metabolic activities, and interactions in these areas. The gut microecology is particularly notable for its extensive biodiversity, with over 1,000 identified microbial species, predominantly anaerobic bacteria belonging to the *Bacteroidetes*, *Firmicutes*, and *Actinobacteria* [13]. Within a unique environment consisting of a mucus layer, intestinal epithelium, and appropriate pH, these microbes and their metabolites, such as short-chain fatty acids, interact with the host and participate in essential physiological processes, including digestive metabolism, immune regulation, and neurostimulation [14,15]. The oral cavity, the second largest microecology, harbors over 770 bacterial species spanning seven major phyla, including *Actinobacteria*, *Bacteroidetes*, and *Firmicutes*. These microorganisms mostly form biofilms on oral mucosal surfaces, salivary interfaces, and gingival plaques. This special colonization pattern, together with parameters like salivary flow and oxygen levels, regulates the structure and function of the oral microecology, assisting in its balance [16,17].

The oral and gut microecosystems are typically distinct, with few shared taxa. However, compromised integrity of the oral and intestinal barriers can enable pathological cross-talk between these niches. Specifically, on the one hand, intestinal microecological imbalance leads to systemic inflammation and weaken the oral mucosal immune function, thereby triggering oral microecological imbalances and causing oral diseases like periodontitis [18,19]; on the other hand, oral microorganisms like *F. nucleatum*, *Porphyromonas gingivalis* (*P. gingivalis*), along with their virulence metabolites, may also breach barrier constraints, translocate, and colonize the gut, further altering the gut microecology [20,21]. The two patterns described above establish a pathologic link to the oral-gut microbiome axis, primarily mediated through two core mechanistic elements: firstly, disruption of physiological differences inherent in different ecological niches, manifested through abnormal changes in environmental parameters such as pH gradient, oxygen partial pressure, and nutrient supply; and second, impairment of theoral-intestinal barrier function due to pathological changes in the body such as bacterial dysbiosis, local inflammatory response, and immune dysfunction [12,22]. This abnormal “oral-gut axis” significantly contributes to gastrointestinal disorders, particularly ulcerative colitis and CRC [3,12].

In recent years, substantial advancements have been achieved in the microbiome investigation of CRC. While the involvement of intestinal microbiota in this disease has been widely elucidated, there is increasing evidence that pathological alterations in the “oral-gut axis”, especially the ectopic colonization of the intestine by oral-derived microorganisms such as *F. nucleatum*, *P. gingivalis*, and their aberrant spread of virulence factors, also play a key role in CRC development. *F. nucleatum* and *P. gingivalis* are abundant Gram-negative obligate anaerobic bacteria in the human oral cavity. Both can penetrate the oral mucosal barrier to enter the bloodstream or reach the gastrointestinal tract, ultimately migrating ectopically to the intestines [3,12]. *F. nucleatum* serves as a bridging microorganism in the oral niche by using surface adhesins like FadA, Fap2, and RadD to co-aggregate with early- and late-colonizing microorganisms [23]. It achieves intestinal colonization through specific recognition and binding to receptors on gut epithelial cells. This process facilitates tumor cell proliferation, immune evasion, genetic and epigenetic alterations, and enhanced chemotherapy resistance, thereby driving CRC development and poor prognosis [7,8]. Previous studies simulating gut colonization of orally derived *F. nucleatum* have confirmed that its surface adhesin binds to E-cadherin on intestinal epithelial cells, activating downstream signaling pathways and promoting the transition from an inflammatory microenvironment to a tumorigenic one. Moreover, intervention with a herbal compound has further demonstrated the targetability of this pathway [24–26]. In contrast, *P. gingivalis* utilizes its acid tolerance and multiple virulence factors – including fimbriae, lipopolysaccharide, outer membrane protein, gingipains, and outer membrane vesicles – to facilitate intestinal colonization [27,28]. Subsequently, it accelerates CRC progression by activating inflammatory pathways and disrupting gut microbiota homeostasis [29,30]. Although the pathogenic traits of these microorganisms have significantly enriched our understanding of the “oral-gut axis”, comprehensively elucidating the pathological mechanisms of this axis will require continued integration of new members and evidence.

Notably, a recent study found that *Peptostreptococcus* coexists within the mucosal microbiota linked to CRC, alongside the aforementioned predominant oral bacteria [9]. In contrast to *F. nucleatum* and *P. gingivalis, Peptostreptococcus*, characterized as Gram-positive anaerobes with spherical or ovoid morphology, was initially isolated and identified by Kluyver and van Niel in 1936. Common strains in the genus include *Peptostreptococcus anaerobius* (*P. anaerobius*), *Peptostreptococcus stomatis* (*P. stomatis*), and *Peptostreptococcus russellii*, among others [31–33], with the first two residing in the oral cavity. This genus produces abundant adhesion proteins [34,35] and can form multilayered biofilms [36]. These traits not only facilitate colonization within the complex oral environment, but also provide a critical advantage for breaching the intestinal mucus barrier and competing for ecological niches upon reaching the gut [37]. As an opportunistic pathogen, multiple studies have demonstrated that *Peptostreptococcus* can promote the development of periodontal disease, inflammatory bowel disease, gastric cancer, and particularly CRC through abnormal proliferation [38–40]. Its substantial pathogenic potential may have long been underestimated, offering new research directions for unraveling the oral-gut microecological crosstalk.

## Association of oral-derived *Peptostreptococcus* with CRC

The National Institutes of Health Human Microbiome Project database indicates that *Peptostreptococcus* is more abundant in healthy individuals’ oral samples than in their fecal ones [41]. Concordantly, analysis of 807 CRC patients’ tumor tissues demonstrated a higher prevalence in the oral cavity [42], implying that it may originate from “passive” oral-gut microbial translocation. Schmidt et al. [6] demonstrated that *P. stomatis* is a key member of the oral-gut transmission route in both healthy individuals and CRC patients. Moreover, the “oral-to-gut score”, which sums the relative abundances of typical oral microorganisms detected in fecal samples, was significantly elevated in CRC [6,43]. A higher score indicates more extensive oral-to-gut microbial transmission and reflects an increased overall colonization level of these microorganisms in the gut. In contrast, *F. nucleatum*, which has been confirmed to share identical strains in the CRC gut and oral cavity, was found to be an occasional transmigrant [44]. Flemer et al. [45] found that CRC patients’ oral and colonic tissues have comparable bacterial networks, including *Peptostreptococcus*, which is commonly identified with oral bacteria like *F. nucleatum* and *P. gingivalis* [10,46]. This finding further supports the propensity of *Peptostreptococcus* to colonize the oral cavity. The current transmission pathways of oral microorganisms along the “oral-gut axis” include both the enteric route and hematogenous routes [47,48]. In antibiotic-treated *APC*^*min/+*^ mice, oral gavage of *P. stomatis/P. anaerobius* demonstrated intestinal mucosal colonization via the enteric route, as validated by spatially resolved tissue localization in colorectal tumors [34,35]. Further studies confirmed that it can compromise intestinal barrier function by reducing tight junction proteins [34] and laying the groundwork for *Peptostreptococcus* enteric translocation in the intestine. In addition, as a periodontal pathogen [49], it may enter the bloodstream and reach the intestine when periodontal pockets are vascularized or gingival ulcers occur. Clinical studies have indicated that this genus can cause bacteremia and increase CRC risk [50], albeit additional validation is required. When *Peptostreptococcus* translocates to the intestine, it will colonize the mucosa and stimulate CRC cells’ malignant proliferation through surface proteins such as Fructose 1,6-bisphosphate aldolase (FBA) [34]. Similarly, *F. nucleatum* also has proteins that aid in mucosal colonization [51,52], suggesting a common mechanism for oral microorganisms’ translocation and colonization. Overall, *Peptostreptococcus* may achieve oral-gut cross-organ transmission via both the enteric and hematogenous routes, potentially initiating CRC by colonizing the intestinal mucosa with surface adhesion proteins. Mounting evidence links this genus and its metabolites in the intestine with CRC [34,53–59].

### Population heterogeneity of *Peptostreptococcus* in CRC patients

**Figure 1.**
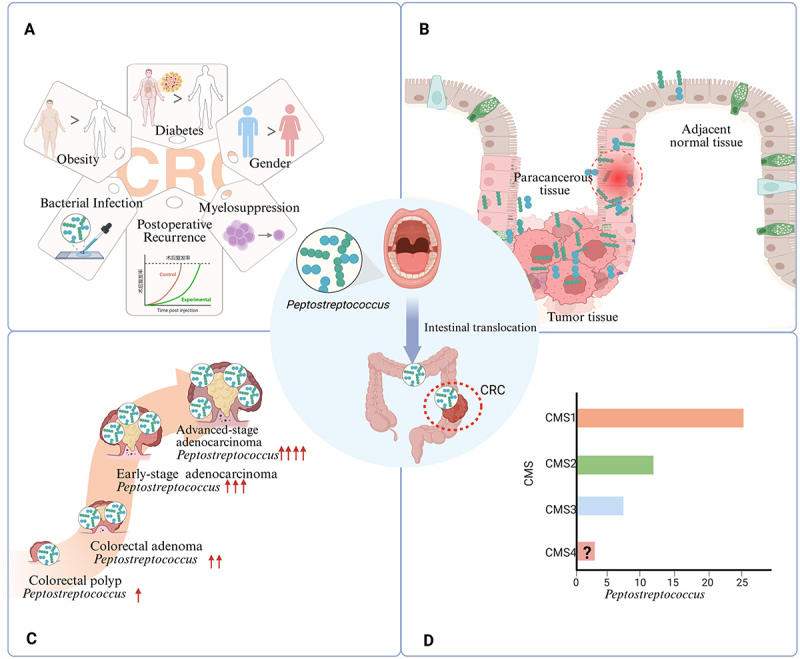
The association between oral-derived *Peptostreptococcus* and CRC. A: population heterogeneity of *Peptostreptococcus* in CRC patients; B: distribution of *Peptostreptococcus* in different spatial locations of CRC tissues; C: dynamic changes of *Peptostreptococcus* during CRC progression; D: distribution characteristics of *Peptostreptococcus* across various molecular subtypes of CRC, particularly its enrichment in CMS1, which is characterized by immune activation and MSI. Graphics created using BioRender.com.

*Peptostreptococcus* is significantly and positively correlated with specific CRC groups (Figure 1(A)). It was noted that this genus in tumor tissues was not only associated with shorter overall survival in CRC patients [60] but also increased the risk of postoperative recurrence [61] and the incidence of myelosuppression following chemotherapy [62], marking it as a poor CRC prognostic indicator. This strong clinical association suggests that oral-derived *Peptostreptococcus* is not merely a “bystander” but may act as a “driver” that actively promotes malignant progression of tumors. Its adverse prognostic effect is likely attributable to its ability to recruit myeloid-derived suppressor cells and suppress CD8^+^ T cell function, thereby fostering an immunosuppressive microenvironment [63,64]. A large cohort study [65] found *P. stomatis* levels rise with body mass index (BMI), interacting with *Faecalibacterium prausnitzii* in obesity to synergistically promote cancer. Furthermore, the genus was shown to be disproportionately enriched in patients with concurrent diabetes mellitus and CRC [66], suggesting its potential mediating role in metabolism-related CRC. This may be explained by the fact that diabetes or high-glucose/high-fat diets can induce or exacerbate intestinal barrier dysfunction [67], creating a favorable ecological niche for the translocation, colonization, and proliferation of oral-derived *Peptostreptococcus*. Additionally, a large cohort study conducted in Hong Kong revealed that bacteremia from this genus increases CRC risk [50]. Yu et al. [68] identified *P. anaerobius* as a notable characteristic of male CRC by third-generation PacBio sequencing. Androgens may disrupt the gut microbiota in CRC patients [69], promoting the overgrowth of opportunistic pathogens such as *Peptostreptococcus*, which in turn exacerbates colonic inflammation and tumor growth. Despite observations indicating that its abundance does not vary significantly with age [70] or geographic location [61], substantial evidence supports the population-specific associations of this genus in CRC.

CMSs: consensus molecular subtypes, ↑ up-regulation. ↓ down-regulation.

In summary, *Peptostreptococcus* represents a key microbial contributor to CRC progression and is significantly enriched in specific high-risk populations, including those with abnormal sex hormone levels and dysregulated glucose and lipid metabolism. Such individuals often exhibit compromised intestinal barrier function, which facilitates the colonization and expansion of oral-derived *Peptostreptococcus*. The observed population heterogeneity in CRC patients further supports the proposed “oral-gut axis” transmission route of this bacterium. As *Peptostreptococcus* continues to reshape the TME, it ultimately contributes to adverse clinical outcomes such as shortened survival, highlighting its potential as a target for early screening and diagnosis in high-risk populations.

### Distribution of *Peptostreptococcus* in different spatial locations of CRC tissues

The distribution of *Peptostreptococcus* in CRC demonstrates considerable spatial heterogeneity (Figure 1(B)). Analysis of enriched bacterial genera in distinct colonic tissues (normal, paraneoplastic, and carcinomatous) of CRC patients determined that *P. anaerobius* was more prevalent in tumor tissues [71]. In vitro adhesion assays demonstrated enhanced binding affinity of this pathobiont to CRC cell lines compared to normal colonic epithelial cells [35]. A recent comparative analysis of 17 shared operational taxonomic units across CRC and colon polyp tissues and clustered based on their abundance in tumor samples, revealed that *P. stomatis* was more prevalent in the mucosa of proximal tumors than distal ones [45]. Moreover, microorganisms in this region are considered not only more pathogenic than those in distal tumor tissues but also closely associated with poor prognosis [72], suggesting that *Peptostreptococcus* may play a critical role in the development and progression of CRC, and could serve as a potential microbial biomarker for prognostic assessment. Further studies showed that *Peptostreptococcus* could disseminate to adjacent normal tissues, with its abundance progressively diminishing from proximal to distal tumor tissues [72]. In addition, *Peptostreptococcus* abundance differed across sample types, being higher in colon mucosa than in fecal samples [73]. Overall, the spatial distribution of *Peptostreptococcus* in CRC exhibited heterogeneity, characterized by enrichment in the proximal tumor mucosa region with a gradual decline toward normal tissues. This mucosa-specific distribution pattern facilitated interactions with intestinal epithelial cells, reinforcing its link to CRC.

### Dynamic changes of *Peptostreptococcus* during CRC progression

The dynamics of *Peptostreptococcus* are intricately linked to CRC progression (Figure 1(C)). Research by Hua et al. [74] has found that *Peptostreptococcus* occurs as a pivotal genus within the bacterial network associated with colorectal adenoma-carcinoma progression, indicating its potential as a biomarker for the diagnosis and treatment of CRC and its precancerous lesions. Furthermore, multiple studies have corroborated that the abundance of *Peptostreptococcus* significantly escalates with disease progression during the transition from colorectal adenoma to cancer [55,75,76]. This upward trend is also evident in the advanced stages of CRC [77,78]. Furthermore, studies have shown that *P. anaerobius* exhibits a higher positive detection rate in CRC patients than in the healthy population, particularly in late-stage CRC [79]. Ge et al. [80] found that *Peptostreptococcus* was more prevalent in high-risk stage III CRC compared to low-risk cases. This pattern of increasing abundance of *Peptostreptococcus* during carcinogenesis and malignant progression may be attributed to the highly hypoxic microenvironment locally formed in tumors [81], which provides ideal conditions for the survival of this obligate anaerobe. Concurrently, the compromised mucosal barrier function associated with disease progression further facilitates its translocation, colonization, and proliferation. More importantly, this genus can actively exacerbate malignant progression by activating cancer-related signaling pathways [34], thereby forming a positive feedback loop. Thus, *Peptostreptococcus* plays a significant role in promoting CRC progression and may serve as a predictive biomarker for disease evolution.

### Distribution characteristics of *Peptostreptococcus* across various molecular subtypes of CRC

The consensus molecular subtypes (CMSs) of CRC are four subtypes (CMS1-CMS4) distinguished by molecular features such as gene expression, with each subtype having unique biological attributes, prognostic implications, and treatment response. It has been shown that tumor-specific microorganisms can be utilized to identify and analyze these subtypes [82]. Based on transcriptome and metagenome sequencing, Purcell et al. [46] discovered that *P. stomatis*, along with *F. nucleatum* and *P. gingivalis*, was highly enriched in CMS1 compared to the other molecular subtypes (Figure 1(D)). CMS1 is defined as an immunogenic subtype with immune response activation and microsatellite instability (MSI) [83]. Further research involving 16S rRNA sequencing of fecal samples from 275 CRC patients and 95 healthy controls revealed that *P. stomatis* and *F. nucleatum* could form a distinct bacterial network, with abnormal enrichment observed in patients with MSI tumors [84]. This bacterial genus is specifically enriched in CMS1-type CRC. On one hand, it may serve as an antigen involved in activating the initial anti-tumor immune response; on the other hand, it can recruit immunosuppressive cells such as MDSCs, tumor-associated macrophages, and tumor-associated neutrophils, thereby shaping an immunosuppressive microenvironment [35,64]. This exacerbates the localized immunosuppressive state in the CMS1 subtype and promotes tumor immune escape. Consequently, targeting *Peptostreptococcus* has emerged as a potential novel strategy to reverse CMS1 tumor immunosuppression and enhance the efficacy of immunotherapies like immune checkpoint inhibitors.

In conclusion, *Peptostreptococcus* colonization in the intestines of CRC patients showed significant specificity across populations, spatial, temporal, and molecular subtypes. Its distribution is closely linked to BMI, gender, and clinical outcomes such as tumor recurrence and post-chemotherapy myelosuppression. Additionally, its abundance varies significantly with spatial locations within CRC, adenoma-carcinoma progression stages, and molecular subtypes (Figure 1, Table 1).

**Table 1.** Studies on the association between *Peptostreptococcus* and CRC.

Research object	Research methods	Sample type	Major finding	Reference
25 healthy controls and 25 CRC patients; 54 healthy controls and 74 CRC patients; 1 healthy control and 1 CRC patient; 490 CRC	16S rRNA gene Metagenome, qPCR LC-MS, IHC	Fecal samples	In CRC patients, there is a notable increase in *Peptostreptococcus*, such as *P. stomatis*, *P. anaerobius*, and their tryptophan metabolites in the gut. Introducing microbial biomarkers improves the sensitivity of fecal immunochemical tests for detecting colorectal lesions.	[54,57–59]
275 CRC and 95 healthy controls	16S rRNA gene		*P. stomatis* was significantly enriched in patients with MSI-type CRC.	[84]
35 healthy controls, 29 colorectal adenomaand 30 CRC; 61 healthy controls, 47 colorectal adenomaand 46 CRC	metagenomics 16S rRNA gene		Compared to the healthy control group and the colorectal adenoma group, the abundance of *P. anaerobius* increased sharply in CRC.	[55,76]
589 CRC and data from published studies (4,439 CRC patients and controls)	16S rRNA gene		The association between *P. anaerobius* and CRC demonstrates strong robustness; it addresses two gaps in CRC microbiome research: quantifying microbiome traits related to cancerous colon changes and identifying microbial factors that may obscure true microbiome-CRC connections.	[85]
522 CRC and healthy controls	Metagenomics, Metabolome		*P. stomatis* shows a positive correlation with BMI in CRC patients; BMI criteria are specific to the Chinese population.	[65]
12 CRC, 12 diabetes-CRC and 12 healthy controls	Metagenomics, Targeted metabolome		The abundance of *Peptostreptococcus* is higher in patients with CRC complicated by diabetes.	[66]
212 healthy controls and 212 CRC	PCR		*P. anaerobius* is the most significant signature bacterium in male CRC.	[68]
460 CRC (262 cases under 50 years old and 198 cases aged 50–88 years)	Shotgun metagenomics		The abundance of *P. stomatis* shows no significant difference across different age groups in CRC.	[70]
41 treatment-naïve CRC cases and 40 non-CRC controls	16S rRNA gene		The association between *Peptostreptococcus* and CRC is comparable between developed and developing countries.	[61]
6 paired normal tissues, 20 paired CRC and adjacent normal tissues, as well as 2 additional unpaired tumors from 2 CRC patients	FISH, 16S rRNA gene	Colon tissue samples	CRC tissues are enriched with invasive biofilms (particularly on right-sided tumors), which are composed of oral pathogens such as *P. stomatis*.	[56]
32 CRC	16S rRNA gene,FISH, qPCR, Metagenomics, Metabolome	Colon tissue samples	*P. anaerobius* is significantly more abundant in CRC tumor tissues than in normal or adjacent non-cancerous tissues.	[71]
34 CRC	16S rRNA gene, PCR	Colon tissue samples	Transcriptomic and metagenomic studies have associated the bacterial species *P. stomatis* with the CRC subtype CMS1.	[46]
338 CRC	16S rRNA gene, qPCR	Mucosal samples	The *Peptostreptococcus* is enriched in advanced-stage CRC; a new gene mutation-based prognostic tool has been created to identify high-risk stage III CRC patients.	[80]
13,096 CRC	Positive bacterial culture	Blood culture samples	*Peptostreptococcus*-related bacteremia increases CRC risk, suggesting microbiota-induced bacteremia as an early CRC warning.	[50]
30 healthy controls and 93 CRC	16S rRNA gene	Saliva samples Fecal samples Subgingival fluid Tumor tissue	*Peptostreptococcus*, *Fusobacterium*, and *Parvimonas* are prevalent oral bacteria associated with CRC.	[53]
51 healthy controls and 52 CRC	16S rRNA gene	Saliva samples Fecal samples	Local oral bacteria may have promoted the initiation of CRC carcinogenesis.	[86]
Six cohorts comprising healthy controls and CRC patients; 98 CRC	qPCR, Metagenomics, IHC, FISH, LC-MS, 16S rRNA gene, FOBT	Saliva samples Fecal samples	The *Peptostreptococcus, like P. stomatis*, is enriched in CRC, and its abundance in both tumor mucosa and adjacent normal mucosa is higher than that in fecal samples.	[34,73]
103 healthy controls, 32 colorectal adenoma and 99 CRC	16S rRNA gene	Oral swab Colonic mucosa Fecal samples	*P. stomatis* is linked to CRC, showing higher abundance in proximal tumor mucosa; a microbial biomarker classifier using oral and fecal microbiota effectively distinguishes CRC and adenomas from healthy individuals.	[45]
Colon tissues from 96 CRC, 82 adenomas and 77 healthy controls; fecal samples from 58 CRC patients and 54 healthy controls	qPCR, Metagenomics, 16S rRNA gene	Colon tissues Fecal samples	*P. anaerobius* levels are higher in CRC patients’ fecal samples and increase from normal tissue to adenoma and CRC.	[75]

## *Peptostreptococcus* in CRC pathogenesis

Although substantial evidence links abnormal *Peptostreptococcus* changes in the “oral-gut axis” to CRC progression, research on its mechanisms remains nascent. This paper reviews existing literature to clarify *Peptostreptococcus*‘s role in carcinogenesis, including tumor cell proliferation, epithelial–mesenchymal transition (EMT) induction, and TME reshaping, aiming to guide future mechanistic investigations.

### Promoting tumor cell proliferation

**Figure 2.**
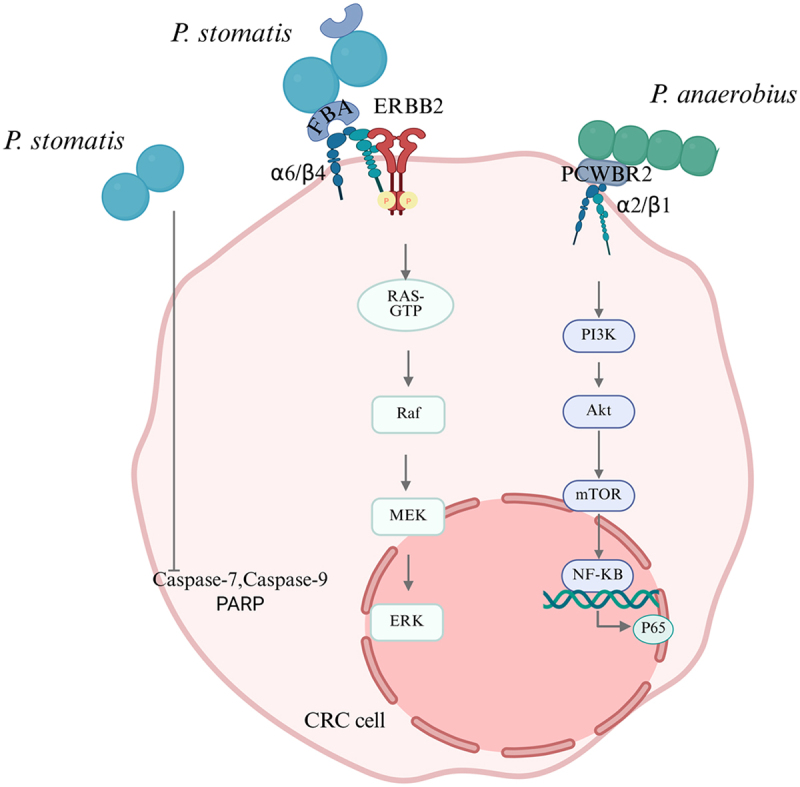
*Peptostreptococcus* promotes tumor cell proliferation. Graphics created using BioRender.com.

Oral-derived *Peptostreptococcus*, upon translocation and colonization in the gut via the “oral-gut axis”, can accelerate tumor progression by either directly promoting tumor cell proliferation or indirectly facilitating it through bacterial surface proteins (Figure 2). Empirical evidence indicates that continuous gavage of *P. stomatis*/*P. anaerobius* dramatically enhances the incidence of colorectal highly heterogeneous hyperplasia, adenomas, and adenocarcinomas in *APC*^*Min/+*^ mice after administration of antibiotics to treat endogenous microorganisms [34, 35]. These findings support the hypothesis that oral microbes translocating through the “oral-gut axis” are sufficient to drive tumorigenesis and tumor development. In vitro, *P. stomatis* stimulated the proliferation of five human CRC cell lines in a time-dependent manner without affecting normal colonic cells. The experimental results revealed that the bacterium up-regulated the expression of the cell cycle markers such as Cyclin D1 and CDK6, and inhibited the cleaved form of key proteins in the mitochondrial apoptotic pathway, including caspase-7, which in turn inhibited CRC cell apoptosis and promoted G1-S cell cycle progression [34].

*P*. *anaerobius*: *Peptostreptococcus anaerobius*; *P. stomatis*: *Peptostreptococcus stomatis*; FBA: Fructose 1,6-bisphosphate aldolase; PCWBR2: putative cell wall binding repeat sequence 2; ERBB2: erb-b2 receptor tyrosine Kinase 2; Raf: rapidly accelerated fibrosarcoma; MEK: mitogen-activated protein kinase; ERK: extracellular regulated protein kinases; PARP: poly ADP-ribose polymerase; PI3K: phosphoinositide 3-kinase; Akt: protein kinase B; mTOR: mammalian target of rapamycin; NF-κB: nuclear factor kappa-B.

Further investigations have identified that bacterial surface proteins are crucial in promoting tumor cell proliferation. Surface proteins of *Peptostreptococcus* possess adhesive capabilities, which serve as an essential foundation for their successful translocation from the oral cavity to the gastrointestinal tract and ultimately facilitate pathogenicity. *P. stomatis* primarily relies on its FBA to bind integrin receptors α6β4 on CRC cells. This specific interaction not only enhances bacterial colonization efficiency on the intestinal mucosa and strengthens bacterial adhesion to cancer cells, but also persistently activates the ERBB2–MAPK signaling pathway, thereby promoting tumor cell proliferation [34]. *P. anaerobius* utilizes its unique surface protein putative cell wall binding repeat sequence 2 (PCWBR2) to interact with integrin receptors α2β1 on CRC cells, activating the PI3K – Akt pathway and upregulating genes like *Itga2*, *Akt1*, and *Nfκb1*, along with an increase in cell proliferation markers [35]. These findings demonstrate that *Peptostreptococcus* species, through their specific surface proteins, enhance their ability to translocate from the oral cavity and ectopically colonize the intestines, thereby creating preconditions for subsequent involvement in CRC progression. Indeed, this mechanism, where related molecules mediate colonization and promote tumor cell proliferation, is also observed in other oral bacteria, although the molecular entities and modes of action may differ. For instance, *F. nucleatum* employs its unique adhesin FadA to bind host E-cadherin, aiding bacterial invasion and activating oncogenic pathways like Wnt/β-catenin; Meanwhile, *P. gingivalis* secretes virulence factors like gingipains to disrupt the epithelial barrier and promote tumorigenesis [87,88]. In addition, *Fusobacterium* has also been shown to enhance CRC cell proliferation and invasion by upregulating microRNAs [89].

### Inducing EMT

Oral-derived *Peptostreptococcus* can induce EMT in tumor metastasis by upregulating mesenchymal markers and transcriptional factors. EMT is a dynamic process in which epithelial cells lose apicobasal polarity to establish contact with neighboring cells, acquire a mesenchymal phenotype, and increase motility [90]. This biological change drives tumor development and metastasis. Specifically, upon translocation to the gut, *P. anaerobius* transforms MC38 cells into a mesenchymal-like structure by inducing IL-23 secretion from MDSCs, boosting cell migration and invasion. Furthermore, this process is accompanied by upregulation of the mesenchymal biomarker *N-cadherin* and transcription factors *such as Snail*, *Slug*, and *Twist* [91]. Notably, this mechanism of MDSC-dependent IL-23 secretion leading to EMT induction appears to be unique to the *Peptostreptococcus* so far. Unlike other oral microbes like *F. nucleatum* and *P. gingivalis*, which influence EMT and CRC metastasis through non-coding RNA regulation and exosome-mediated gene transfer [92,93]. Moreover, as obligate anaerobes capable of surviving in the hypoxic oral environment, *Peptostreptococcus*’s inherent metabolic traits can be reactivated and utilized upon translocation to the gut, particularly within the hypoxic TME of CRC. In addition, *P. anaerobius* utilizes tryptophan metabolites in the intestinal environment to promote colorectal carcinogenesis through activation of the aryl hydrocarbon receptor [59]. Sustained activation of this receptor can downregulate E-cadherin expression and enhance tumor cell migration [94], further suggesting its potential role in regulating EMT. Nonetheless, the molecular mechanism by which *Peptostreptococcus* influences EMT-regulated metastasis remains incompletely understood. More research is required to explore its functional targets and signaling pathways in CRC metastasis, which could reveal new microbe-host interaction mechanisms and inform targeted microbial therapeutics.

### Reshaping the TME

TME represents a complex ecosystem of tumor cells, immune cells, and extracellular matrix, which is essential for the malignant progression of CRC.

#### Recruiting tumor-infiltrating immune cells

**Figure 3.**
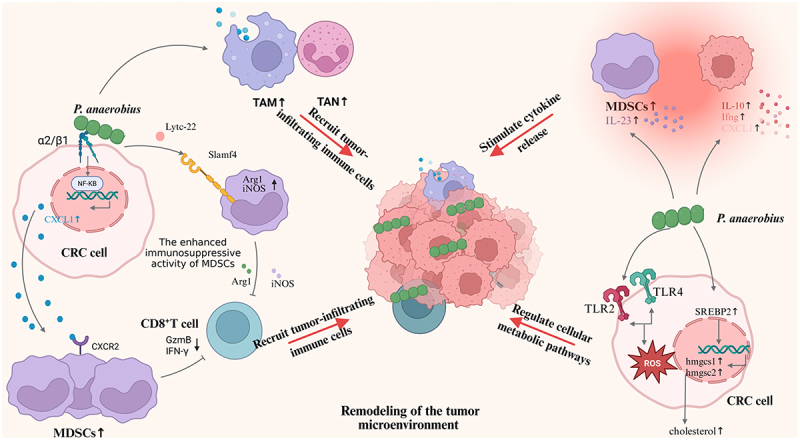
The effect of *Peptostreptococcus* on TME. Graphics created using BioRender.com.

Oral-derived *Peptostreptococcus* can recruit tumor-infiltrating immune cells while inhibiting anti-tumor immune-responsive lymphocytes, resulting in a tumor-promoting immune microenvironment (Figure 3). The oral cavity serves as the immune system’s first defense. To adapt to this unique immune environment, *Peptostreptococcus* may have evolved sophisticated strategies to evade host immune surveillance. Upon translocation to the gut, these bacteria may become key drivers of tumor immune escape. Specifically, *P. anaerobius* inhibits functional CD8^+^ T cells infiltration by activating the integrin α2β1-driven NF-κB signaling pathway in CRC cells, releasing chemokine ligand 1 (CXCL1) to interact with transmembrane receptors on MDSCs, and facilitating MDSCs migration into the TME [64]. Further research has revealed that *P. anaerobius* not only attracts MDSCs to the TME [91] but also secretes a functional protein that interacts directly with MDSCs’ transmembrane receptors. This interaction stimulates the expression of arginase 1(Arg1) and inducible nitric oxide synthase (iNOS), which confer immunosuppressive activity to MDSCs and suppress CD8^+^ T cell immune function [64]. Moreover, tumor-associated macrophages and neutrophils are considerably overrepresented in the CRC mice’s intestines [35]. Although other oral bacteria like *F. nucleatum* and *P. gingivalis* also recruit tumor-associated immune cells to influence the TME, current studies indicate that *F. nucleatum* primarily promotes the proliferation of MDSCs [95], while *P. gingivalis* stimulates MDSCs to activate the NLRP3 inflammasome [30]. In contrast, *Peptostreptococcus* not only recruits MDSCs but also secretes specific functional proteins that bind to them, thereby driving the immunosuppressive functions of MDSCs. This dual regulatory mechanism on MDSCs represents a distinguishing feature that sets it apart from the aforementioned microorganisms. The abnormal immune cells recruited by *Peptostreptococcus* are pathologically active and exceedingly immunosuppressive, which is linked to poor patient outcomes in CRC [63], suggesting their potential as biomarkers for poor prognosis.

TAM: tumor-associated macrophage; TAN: toumor-associated neutrophile; Slamf4: signaling lymphocyte activation molecule family member 4; Arg1: arginase 1; iNOS: inducible nitric oxide synthase; CXCL1: chemokine ligand 1; MDSCs: myeloid-derived suppressor cells; *P. anaerobius*: *Peptostreptococcus anaerobius*; *P. stomatis*: *Peptostreptococcus stomatis*; SREBP2: sterol regulatory element-binding protein 2; CXCR2: c-x-c motif chemokine receptor 2; NF-κB: nuclear factor kappa-B; GzmB: granzyme B; IFN-γ: Interferon-γ; TLRs: toll-like receptors; ROS: reactive oxygen species; hmgcs: 3-hydroxy-3-methylglutaryl-coa synthase. ↑up-regulation. ↓down-regulation.

#### Stimulating cytokine release

Oral-derived *Peptostreptococcus* also increases the expression of cytokines such as chemokines and interleukins in TME, thereby promoting CRC development (Figure 3). As a key pathogen of periodontitis [38], *Peptostreptococcus* can maintain its pro-inflammatory nature when it disseminates to the gut via the “oral-gut axis” and colonizes there. In CRC mice treated with *P. anaerobius*, tumor tissues showed higher IL-23 levels [91], indicating that this bacterium may regulate CRC through particular cytokine pathways. In vitro investigations confirmed that *P. anaerobius* also enhanced CXCL1 expression in Caco-2 cells [64]. In an *APC*^*min/+*^ mouse model, *P. anaerobius* also upregulated pro-inflammatory cytokines, including *IL-10*, *Ifng*, and *Nfκb1* [35]. Cytokine dysregulation can lead to cancer progression through persistent inflammation and immune evasion [96, 97], with NF-κB pathway activation possibly playing a role. Although current evidence indicates that this genus can reshape TME via cytokine modulation, the exact mechanisms require further study.

#### Regulating tumor cell metabolism

Tumor cells can survive and proliferate in nutrient-poor environments by efficiently acquiring nutrients [98]. In addition to various tumor-infiltrating immune cells and cytokines, tumor cell metabolic pathways also affect TME (Figure 3). In contrast to existing studies that predominantly focus on *F. nucleatum*’s impact on tumor cell glycolytic [99], *Peptostreptococcus* primarily influences TME through cholesterol metabolism. Studies have shown that hypercholesterolemia can contribute to periodontitis [100], suggesting *Peptostreptococcus*, a key periodontitis pathogen, may modulate cholesterol metabolism initially acquired in the oral cavity and subsequently exert effects in the gut via the “oral-gut axis”. Gene analysis of human CRC cells treated with *P. anaerobius* showed significant effects on cholesterol-related gene expression [35]. Tsoi et al. [75] discovered that exposing human CRC cell lines to *P. anaerobius* dramatically elevated levels of sterol regulatory element-binding protein 2 (SREBP2) and total cholesterol, which is a transcription factor involved in cholesterol biosynthesis and uptake; whereas inhibiting SREBP2 reduced cholesterol in CRC cells, implying that the bacterium promotes cholesterol synthesis in tumor cells via SREBP2; in addition, *P. anaerobius* interacts with transmembrane proteins – Toll-like receptor 2/4 (TLR2/4) on CRC cells, increasing reactive oxygen species levels and thus enhancing cholesterol biosynthesis. The dysregulation of cholesterol metabolism exacerbates intratumoral microbial imbalance, creating a microenvironment conducive to oral microorganism colonization and accelerating tumor progression.

In summary, oral-derived *Peptostreptococcus* can affect TME by recruiting tumor-infiltrating immune cells, suppressing lymphocytes involved in anti-tumor immunity, increasing cytokine expression, and modulating tumor cell metabolic pathways (Figure 3). Although *Fusobacterium* and *Porphyromonas* share some pro-tumor phenotypic similarities, current evidence reveals that the molecular mechanisms and pathways employed by *Peptostreptococcus* are distinct. Although less researched than *F. nucleatum* and *P. gingivalis*, *Peptostreptococcus* could be a significant emerging target for study. In-depth exploration of its role could enhance our understanding of the complexity of microbial carcinogenesis through the oral – gut axis and may provide a novel theoretical basis and potential targets for future precision interventions targeting specific oncogenic microbiota.

## Techniques for investigating the translocation of microorganisms along the “oral-gut axis”

**Figure 4.**
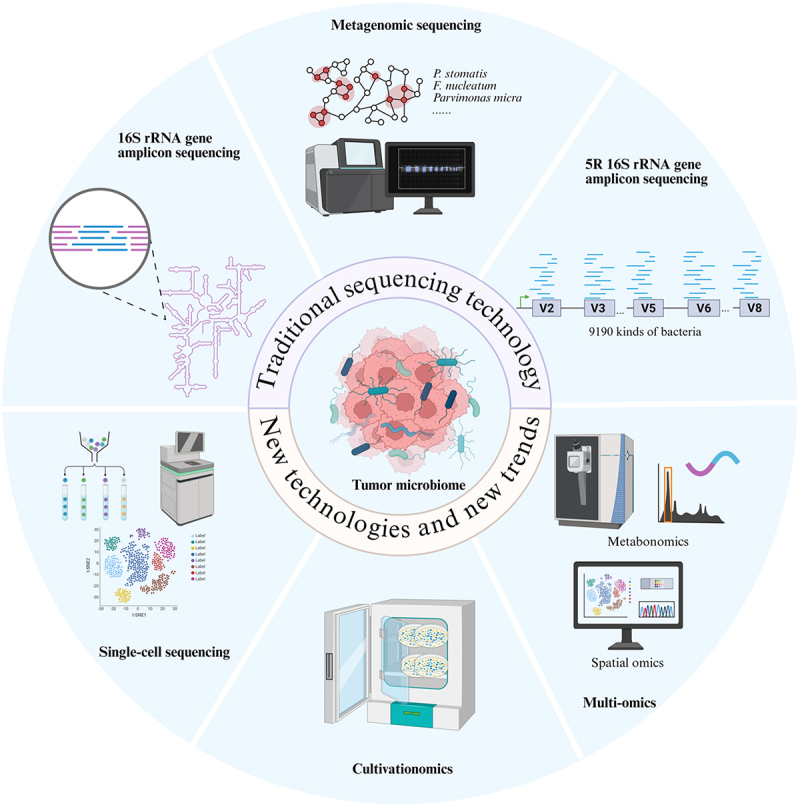
Research techniques of microorganisms’ translocation along the “oral-gut axis”. Graphics created using BioRender.com.

Recent advancements in research, facilitated by NGS, have significantly enhanced our understanding of how microorganisms translocated through the “oral-gut axis” relate to tumorigenesis. These technologies surpass traditional methods, allowing for high-throughput detection and precise identification of tumor-associated microorganisms (Figure 4).

### Classical sequencing technologies

#### 16S rRNA gene sequencing

16S rRNA gene sequencing is an affordable and precise technique for examining microbial communities by targeting specific gene fragments. It is commonly used to identify and quantify tumor-associated microorganisms [101]. This technique has been widely employed to investigate the associations and transmission mechanisms between the oral and gut microbiomes. For instance, sequencing of saliva and intestinal fluid samples showed reduced diversity of oral-derived *Fusobacterium* in the gut of inflammatory bowel disease patients [102], suggesting selective translocation during microbial migration across ecological niches, potentially mediated by the oral-gut barrier. Recently, Conde-Pérez et al. [53] demonstrated that CRC patients had significantly higher levels of oral microorganisms like *Peptostreptococcus*, *Fusobacterium*, and *Parvimonas* in their fecal samples compared to non-CRC individuals using this method, providing preliminary evidence for microbial transmission along the “oral-gut axis”.

#### Metagenomic sequencing

Metagenomic sequencing involves high-throughput sequencing of all microbial genomic DNA in a sample without PCR amplification, enabling species-level analysis of tumor-associated microorganisms’ composition and genes. This technique effectively identifies microbial overlap between the oral and gut [103]. Combined with phylogenetic tree analysis, it further allows exploration of the oral origins of microorganisms at the strain level [53], providing additional evidence for the oral-gut transmission of CRC-associated microbes. Moreover, metagenomic sequencing has revealed that *Peptostreptococcus* is closely associated with multiple cancer-related metabolic pathways [76], thereby strengthening the mechanistic understanding of how translocated oral microbes may contribute to colorectal tumorigenesis at a functional level.

While the two sequencing technologies are common in microbial research, they fail to strike a compromise between high resolution and low cost, limiting their application to tumor tissues and blood samples with minimal microbial presence or substantial host DNA contamination. To address this, Nejman et al. [104] developed 5 R 16S rRNA gene sequencing in 2020, which enhanced bacterial species detection to about 68% of full-length 16S sequences by using multiplexed PCR to amplify and sequence five regions (V2, V3, V5, V6, and V8) on the 16S rRNA gene. This method significantly reduced assay costs and showed excellent compatibility with samples, even for formalin-fixed paraffin-embedded (FFPE) samples with degraded nucleic acids, allowing for reliable species-level analysis. This technology thereby provides a highly effective research tool for accurately tracing the transmission of microorganisms from the oral cavity to the gut.

### Novel technologies and trends

#### Microbial single-cell sequencing

Advancements in microbial single-cell sequencing are transforming microbiome research by addressing previous NGS limits in resolving colony heterogeneity and detecting rare species. This technology provides strain-level resolution, identifies unculturable or unknown species, and offers evidence of strain identity for microbial migration from oral to gut environments by analyzing individual cells. In 2022, Zheng et al. [105] introduced a high-throughput single-cell genome sequencing method using microfluidics, allowing for detailed characterization of complex microbial communities. Further developments include single-cell analysis of host-microbiome interactions (SAHMI), which reconstructs microbial signals through data denoising [106], and invasion-adhesion-directed expression sequencing (INVADE), which identifies intracellular bacteria by targeting specific 16S rRNA gene regions [107]. These innovations enhance the study of tumor-microbe interactions at the single-cell level. scRandom-seq [108] and smRandom-seq [109] further integrate random primer amplification with cross-species algorithms to capture host and microbial RNAs, allowing transcriptional profiling of bacteria – phage interactions. These tools provide powerful means to investigate functional heterogeneity of microbes in the TME and adaptive responses of orally derived microbes migrating to the gut at single-cell resolution. Using single-amplified genomes derived from single-cell genomic sequencing, strain identity between oral and fecal samples has been observed, offering cellular-level evidence of microbial translocation from the oral cavity to the gut [110]. These technological breakthroughs not only overcome traditional sequencing’s cell mixing issues but also reveal the microbial community interactions in terms of gene expression, functional heterogeneity, and genetic variation, establishing a precise research framework for exploring host-microbial interactions and therapeutic targets in TME. Moreover, these approaches significantly advance our understanding of microbial transmission routes such as the “oral – gut axis” and “oral – lung axis”, as well as their mechanisms in disease pathogenesis.

#### Culturomics

NGS has broadened our understanding of bacterial taxonomy and function, while also highlighting the necessity of using culturomics to obtain physiologically active microbial strains from specific niches. In the study of the “oral-gut axis”, culturomics provides an irreplaceable experimental foundation for validating microbial translocation hypotheses and elucidating strain functions by supplying actionable live bacterial resources. Unlike traditional isolation methods reliant on single culture conditions and phenotypic observations, microbial culturomics identifies isolated strains by designing a multifaceted culture system (including medium components optimization, oxygen concentration gradient control, temperature regulation, and host microenvironment simulation) and combining it with MALDI-TOF mass spectrometry, full-length 16S rRNA gene sequencing, and other technologies [111]. Utilizing culturomics, Conde-Pérez et al. [53] successfully isolated *Parvimonas micra* from gingival crevicular fluid and adenocarcinoma samples of CRC patients, providing key evidence supporting microbial translocation from the oral cavity to the gut and its involvement in intestinal pathology. Recent advancements in novel culture strategies have accelerated their breakthrough in the limitations in studying complex microbial communities. The CAMII platform enhances microbial isolation by combining colony morphology with genomic data, enabling targeted genus screening [112]. The smart-chip gut system integrates microfluidic chips with environmental controls to co-culture microorganisms and host cells, aiding the isolation of difficult-to-cultivate microbes like anaerobes [113]. Future microbial culture methods should integrate multidisciplinary platforms and high-throughput sequencing to disclose additional “microbial dark matter” and its metabolic roles, aiding in understanding microbial-host interactions within the TME in the context of the “oral-gut axis”.

#### Multi-omics joint analysis

Single-cell microbiomics reveals microbial population heterogeneity, and culturomics overcomes the limitations of traditional non-culturable microorganisms, while multi-omics integrates multidimensional data to systematically analyze microbial functions and their interactions with the environment or host, providing a comprehensive framework for investigating the translocation and pathogenic mechanisms of microorganisms along the “oral-gut axis”. Metabolomics/metaproteomics, which employs nuclear magnetic resonance spectroscopy or mass spectrometry, detects microbial metabolites/proteins to infer microbial activity and host interactions [114,115]. Li et al. [65] found that increased *P. stomatis* in obesity-related CRC patients disrupts lipid metabolism, aggravating intestinal conditions and tumor progression by integrating metagenomic, metabolomic, and random forest analyses. Their findings provide functional evidence reinforcing the mechanistic understanding of the “oral-gut axis” in disease pathogenesis. At the spatial resolution level, immunohistochemistry (IHC) localizes specific protein expression via antibody-antigen reactions, while fluorescence in situ hybridization (FISH) pinpoints microorganisms using fluorescent nucleic acid probes. Both are often used in conjunction with metagenomics and metabolomics to analyze the spatial distribution of microorganisms like *Peptostreptococcus* in tumor tissue samples [34], [71]. The advent of innovative spatial genomics technologies has provided greater resolution analytical tools for microbe-host interaction studies within the “oral-gut axis”. For instance, examining FFPE breast cancer samples with GeoMx whole transcriptome and immune profiling atlases revealed molecular differences between *F. nucleatum*-colonized and non-colonized regions, showing how proliferative regions reshape the microenvironment by modulating RNA/protein expression linked to proliferation, migration, and invasion [116]. Integrated multi-omics approaches enable the tracing of microbial transmission along the oral-gut axis at both taxonomic/functional levels of microorganisms and genomic/metabolomic levels of the host. These approaches further allow in-depth analysis of microbe-tumor interactions, yielding novel biomarkers for precise cancer diagnosis and treatment, personalized intervention targets, as well as comprehensive mechanistic insights.

## Challenges and prospects

### Clarifying the causal relationship between *Peptostreptococcus* and CRC development

Although the association between *Peptostreptococcus* and CRC is evident, the causal relationship remains to be elucidated. Current evidence supporting the oral origin of *Peptostreptococcus* in CRC tissues primarily relies on correlative studies, such as differential enrichment in oral versus fecal samples and co-aggregation with common oral microbes. However, these associations do not definitively establish an oral origin or a causal link. The “alpha-bug” hypothesis [117] emphasizes the pro-cancer effects of specific pathogens by disrupting the host microenvironment and “crowding out” probiotics. The “driver-passenger” model [118] proposes that native gut microbes act as “drivers,” inducing DNA damage in intestinal epithelial cells and creating conditions favorable for “passenger” bacteria, such as *F. nucleatum*, to thrive. These passengers may eventually dominate in response to alterations in TME. Studies using antibiotic-depleted gut microbiota in *APC*^*Min/+*^ mice [34,35] have attempted to validate *Peptostreptococcus* as a CRC-promoting factor. However, these models, which typically involve direct oral gavage or intraperitoneal injection of bacteria, fail to recapitulate the natural migration route from the oral cavity to the gut. Thus, it does not directly demonstrate an oral origin of CRC-associated *Peptostreptococcus*. Moreover, antibiotic treatment itself disrupts gut ecological integrity and alters local immune conditions, making it impossible to distinguish between direct carcinogenic effects of the bacteria and indirect niche effects – that is, whether opportunistic expansion into dysbiosis-induced vacant niches leads to selective growth advantages, indirectly exacerbating microenvironment dysregulation and disease progression. Additionally, significant differences in microbiome structure and immune systems between animal models and humans complicate the extrapolation of results. Therefore, it remains unclear whether *Peptostreptococcus* dysbiosis is a cause, consequence, or bidirectional contributor in CRC pathogenesis. Future studies should employ metagenomic and phylogenetic analyses to compare paired oral and tumor samples from CRC patients at the strain level, assessing genetic homology and evolutionary relationships between *Peptostreptococcus* isolates from both sites. Additionally, establishing in vitro co-culture models, organoid platforms, and using genetically labeled strains to track oral-to-gut microbial translocation in humanized animal models will be crucial to clarify the causal role of oral-derived *Peptostreptococcus* in CRC development and progression.

### In-depth investigation of the mechanisms of *Peptostreptococcus* during CRC progression

Advancements in high-throughput sequencing have rekindled interest in *Peptostreptococcus*‘s role in CRC, but most studies remain observational without in-depth mechanistic evidence. As summarized above and indicated in recent studies, bacteria transferred via the “oral-gut axis” exhibit similar transmission routes and CRC-promoting mechanisms. Although the specificity of their association with oral sources requires further validation, focusing on *Peptostreptococcus*’s unique characteristics may provide direction for in-depth exploration of its mechanistic roles. (1) *P. gingivalis* is acid-resistant [28] against host defense mechanisms such as gastric acid, along with *F. nucleatum*, which promotes intestinal colonization by releasing virulence factors such as extracellular vesicles, lipopolysaccharides, and fimbriae [119]. Given that *Peptostreptococcus* can be passively transported via the oral-gut route, further studies should examine whether it possesses similar or unique colonization traits. (2) *F. nucleatum* induces non-coding RNA and host DNA damage [120]. Since *Peptostreptococcus* is significantly enriched in MSI tumors, further studies should examine whether it possesses similar or unique colonization traits. (3) The relative increase in oral bacteria abundance after trans-intestinal migration marks the depletion of other intestinal bacteria [121]. Given that *Peptostreptococcus*, along with other oral bacterial species, is enriched in metastatic CRC tissues [60] and increases intestinal permeability [34], further studies should assess whether this genus directly modulates the gut microbial structure and distinctly influences intestinal ecology compared to other oral bacteria, thereby promoting CRC progression. Meanwhile, different *Peptostreptococcus* species/strains might compete or cooperate nutritionally and ecologically in oral and intestinal habitats. The impact of these interactions on their abundance, virulence, colonization of CRC, and overall microecology is unknown and requires experimental validation. (4) *F. nucleatum* can alleviate CD8^+^ T cell depletion and enhance immunotherapy efficacy in CRC [122]. In contrast, current research on *Peptostreptococcus* mainly focuses on its tumor-promoting effects, with limited attention to its therapeutic potential. Further studies should investigate whether it can similarly enhance responses to immunotherapy, radiotherapy, or chemotherapy in CRC. For instance, integrated in vitro and in vivo models could help analyze its synergistic effects with these therapies. It is important to note that, while leveraging research on other oral pathogens, emphasis should be on *Peptostreptococcus*’s unique biological features to more comprehensively elucidate its mechanisms in CRC. In addition, most existing investigations predominantly rely on animal models and tumor cell/organoid-microbe co-culture systems to investigate microbiota-host interactions, which fail to replicate the host’s complex microenvironment. Future research should incorporate more reliable disease models and prospective clinical cohorts to thoroughly define *Peptostreptococcus*’s role in CRC pathogenesis.

### Expanding the clinical utility of oral-derived microorganisms as CRC markers

Microorganisms of oral-derived have the potential as biomarkers for CRC due to their noninvasive collection and link to the “oral-gut axis”. However, challenges in sample selection, detection methods, and data interpretation hinder their clinical application. In terms of sample selection, current noninvasive CRC screening mainly relies on fecal occult blood tests, which are less sensitive to early lesions. In contrast, oral swabs capture a broader spectrum of CRC-related flora, like *Peptostreptococcus*, which exhibits high specificity for patients with CRC and polyps but has a sensitivity of only 50%–70%. However, when combined with fecal microbial data, the sensitivity can be increased to 76%–88% [45], highlighting the potential of an oral-gut microbiome combined strategy to enhance screening sensitivity. Moreover, oral swab testing is a noninvasive, straightforward, cost-effective method with high patient compliance. Its integration with fecal microbial data may provide a new approach for early screening in high-risk populations, which could be further validated through multi-center, prospective clinical trials in the future. It is also essential to fully account for potential confounding factors like diet, medication, and geographic or population differences that may influence the composition of oral and gut microbiota. This is critical for developing diagnostic models with strong generalizability. At the detection level, current studies focus on genus-level analyses due to sequencing resolution limits and strain selection challenges. Future research should employ high-resolution technologies, such as single-cell transcriptome sequencing, alongside spatial multi-omics technologies to thoroughly assess the impact of oral microorganisms on hosts at the strain or subspecies level. In CRC research, it is necessary to analyze the dynamic spatial and temporal changes of microorganisms by sampling at various intervals to establish surveillance models, quantify flora fluctuations with disease course, and improve sample processing and sequencing methods to minimize host DNA interference. While microbiome research grapples with large dataset complexities, emerging bioinformatics techniques show promise. For example, “MintTea” integrates complex datasets and has connected *Peptostreptococcus* to late-stage CRC [123]. Additionally, machine learning algorithms demonstrate the capability to accurately identify CRC-specific biomarkers and host-colony interactions through deep mining of high-dimensional data, considerably improving the efficacy of CRC early screening [124]. This highlights that AI-driven big data integration represents a pivotal strategy to decode microbiome complexity and advance precision oncology.

### Developing targeted intervention strategies against oral-derived microorganisms

Current treatments for CRC caused by *Peptostreptococcus* abnormalities include berberine, which inhibits the genus’s amino acid metabolism [125], and multi-epitope vaccines that induce an immune response against the genus’s surface proteins [126]. Emerging therapeutic strategies for oral-derived microorganisms, such as synthetic antimicrobial peptides targeting bacterial surface proteins [127] and delivery systems based on genetically engineered phage combined with nanoparticles/liposomes [128], may provide direction for targeting the treatment of *Peptostreptococcus*. However, there remains a lack of compelling in vivo experimental evidence to validate the efficacy and safety of the aforementioned strategies, which represents a critical gap that must be addressed in the future translational research. Notably, these microbial modulation strategies may hold potential as adjuvants to cancer therapy, as certain oral-derived microbes, like *Peptostreptococcus*, can recruit MDSCs and confer resistance to anti-PD-1 therapy. Therefore, future studies should investigate combining targeted microbial strategies with immune checkpoint inhibitors to boost anti-tumor responses and overcome resistance. Furthermore, with advancing insights into the mechanisms of virulence factors of oral-derived microbes, such as FBA and PCWBR2 in *Peptostreptococcus*, using CRISPR-Cas to target specific virulence genes offers potential for precision microbiome medicine. Nonetheless, critical issues such as delivery efficiency, safety, and off-target effects of this approach in vivo require extensive further investigation. Leveraging the “oral-gut axis”, future therapies could combine oral microbiota modulation with intestinal ecological reconstruction to address the limitations of single-site interventions. For example, supplementing oral probiotics can inhibit harmful bacteria in the oral cavity and prevent their spread to the gut, while fecal transplantation utilizing donor microbes rich in beneficial bacteria and low in pathogens helps restore healthy gut flora balance. In addition, maintaining oral hygiene and regular dental care can also prevent oral bacteria-related intestinal issues. Oral-derived microorganisms hold promise for therapeutic development, but their unknown adverse effects necessitate further clinical research for safety and efficacy.

## Conclusion

The “oral-gut axis” is an important entry point for understanding how the human microbiome systematically influences host health. Over the past decade, substantial progress has been made in unraveling the implications of dysbiosis within this axis for CRC pathogenesis. This review teases apart the interplay between oral and gut microbiota in CRC development, focusing on *Peptostreptococcus*’s role in CRC progression. We synthesize evidence demonstrating that *Peptostreptococcus* employs surface adhesion proteins to colonize the intestinal mucosa, thereby promoting tumor cell proliferation, inducing EMT, and reshaping the TME. In addition, the genus is expected to serve as a diagnostic biomarker and potential therapeutic target for CRC. Future research should prioritize integrating clinical data with human cohort studies and preclinical animal models, leveraging advanced multi-omics technologies to delineate the molecular mechanisms through which *Peptostreptococcus* influences CRC progression via the “oral-gut axis”. Such efforts will facilitate the development of safe, precise microbial modulation strategies, offering novel avenues for CRC prevention, diagnosis, therapy, and prognosis.

## Data Availability

Data sharing is not applicable to this article as no data were created or analyzed in this study.

## Abbreviations

Arg1: Arginase 1
BMI: Body mass index
CRC: Colorectal cancer
CMSs: Consensus molecular subtypes
CXCL1: Chemokine ligand 1
EMT: Epithelial-to-mesenchymal transition
FFPE: Formalin-fixed paraffin-embedded
FISH: Fluorescence in situ hybridization
*F. nucleatum*: *Fusobacterium nucleatum*
FBA: Fructose 1,6-bisphosphate aldolase
iNOS: Inducible nitric oxide synthase
INVADE: Invasion-adhesion-directed expression sequencing
IHC: Immunohistochemistry
MSI: Microsatellite instability
MDSCs: Myeloid-derived suppressor cells
NGS: Next-generation sequencing
*P. gingivalis*: *Porphyromonas gingivalis*
*P. anaerobius*: *Peptostreptococcus anaerobius*
*P. stomatis*: *Peptostreptococcus stomatis*
PCWBR2: Putative cell wall binding repeat sequence 2
SREBP2: Sterol regulatory element-binding protein 2
SAHMI: Single-cell analysis of host microbiome interactions
